# Retraction Note: Day-ahead electricity price forecasting using WPT, VMI, LSSVM-based self adaptive fuzzy kernel and modified HBMO algorithm

**DOI:** 10.1038/s41598-022-06630-9

**Published:** 2022-02-10

**Authors:** Rahmad Syah, Mohammad Rezaei, Marischa Elveny, Meysam Majidi Nezhad, Dadan Ramdan, Mehdi Nesaht, Afshin Davarpanah

**Affiliations:** 1 Data Science & Computational Intelligence Research Group, Universitas Medan Area, Medan, Indonesia; 2 Computer Science and Engineering Department, University of Texas at Arlington, Arlington, USA; 3 Data Science & Computational Intelligence Research Group, Universitas Sumatera Utara, Medan, Indonesia; 4 Department of Astronautics, Electrical and Energy Engineering (DIAEE), Sapienza University of Rome, 00184 Rome, Italy; 5 DS & CI Research Group, Universitas Medan Area, Medan, Indonesia; 6 Optimisation and Logistics Group, School of Computer Science, University of Adelaide, Adelaide, Australia; 7 Department of Mathematics, Aberystwyth University, Aberystwyth, SY23 3FL UK

Retraction of: *Scientific Reports* 10.1038/s41598-021-96501-6, published online 30 August 2021

The Editors have retracted this Article.

Following publication of this Article, a number of overlaps were identified between the data presented in Tables 2, 3 and 8, and the previous literature^1–3^. In addition, concerns have been raised about the relevance of the references provided, and accuracy of the authorship contributions. The authors were unable to provide a revised version with valid referencing, and unambiguously establish the veracity of the authorship contributions. Given these concerns the Editors no longer have confidence in the study.

Rahmad Syah, Marischa Elveny, Mehdi Nesaht, and Afshin Davarpanah disagree to this retraction. Mohammad Rezaei, Meysam Majidi Nezhad, and Dadan Ramdan have not responded to any correspondence from the Editors about this retraction.
